# Acute otitis media-related facial nerve palsy in a child: a case report and a literary review

**DOI:** 10.1186/s13052-022-01405-4

**Published:** 2023-01-14

**Authors:** Massimo Luca Castellazzi, Sara Torretta, Giada Maria Di Pietro, Annaclara Ciabatta, Pasquale Capaccio, Luca Caschera, Paola Marchisio

**Affiliations:** 1 Fondazione IRCCS Ca’ Granda Ospedale Maggiore Policlinico, Pediatric Emergency Department, Via Commenda 9, 20122 Milan, Italy; 2 Fondazione IRCCS Ca’ Granda Ospedale Maggiore Policlinico, Milan, Italy; 3 Department of Specialistic Surgical Sciences, University of Milan, Milan, Italy; 4 Fondazione IRCCS Ca’ Granda Ospedale Maggiore Policlinico, Pediatric Highly Intensive Care Unit, Milan, Italy; 5 Department of Biomedical, Surgical, and Dental Sciences, University of Milan, Milan, Italy; 6 Fondazione IRCCS Ca’ Granda Ospedale Maggiore Policlinico, Neuroradiology Unit, Milan, Italy; 7 Department of Pathophysiology and Transplantation, University of Milan, Milan, Italy

**Keywords:** Facial nerve palsy, Acute otitis media, Epstein-Barr virus, Children

## Abstract

**Background:**

Acute otitis media has become a rare cause of facial palsy in children. A high index of suspicion is essential to achieve the diagnosis and to properly treat this condition to avoid permanent neurological sequelae.

**Case presentation:**

A case of acute otitis media-related facial nerve palsy in an 18 months-old child is described and a review of the recent literature about the clinical presentation, diagnosis, and management of this condition is performed.

**Conclusions:**

Facial paralysis is an uncommon complication of acute otitis media that requires appropriate care. As highlighted in our report, the treatment of facial nerve palsy secondary to otitis media should be conservative, using antibiotics and corticosteroids. The role of antiviral is still a matter of debate. Myringotomy and a ventilation tube should be added when spontaneous perforation of the tympanic membrane is not present. More aggressive surgical approach should be considered only when there is no significant improvement.

## Background

Acute otitis media (AOM) is one of the most common infectious diseases in children [1]. Acute mastoiditis is the most frequent AOM complication. More severe complications such as meningitis, subperiosteal, epidural, or intracerebral abscesses may occur albeit uncommon [2]. Facial nerve paralysis secondary to concurrent AOM is also possible, even if it is rarely reported in children due to the use of broad-spectrum antibiotics, with only 0.005% of patients developing this condition [3]. The rarity of facial nerve palsy makes the treatment approach a current matter of debate, specifically the need for surgical intervention [4]. Moreover, the pathogenesis of this condition is still unclear, and although AOM is associated with a bacterial etiology in approximately two-thirds of cases, recent studies have documented an increasingly important role of viruses in the pathology [5].

In this context, facial nerve palsy with AOM associated with Epstein-Barr virus (EBV) infection has been rarely observed [5, 6]. Here, we describe a case of a previously healthy male child with acute onset of AOM-related facial nerve palsy associated with recent EBV infection. Furthermore, a review of the recent literature on diagnosis and treatment of AOM-related facial nerve palsy is performed.

## Case presentation

A previously healthy, fully immunized, 18-month-old boy was admitted to our pediatric emergency department for an acute onset of left-side facial palsy. He was on oral antibiotic treatment with amoxicillin (80 mg/kg/day) for fever and bilateral AOM since the day before.

**Fig. 1:**
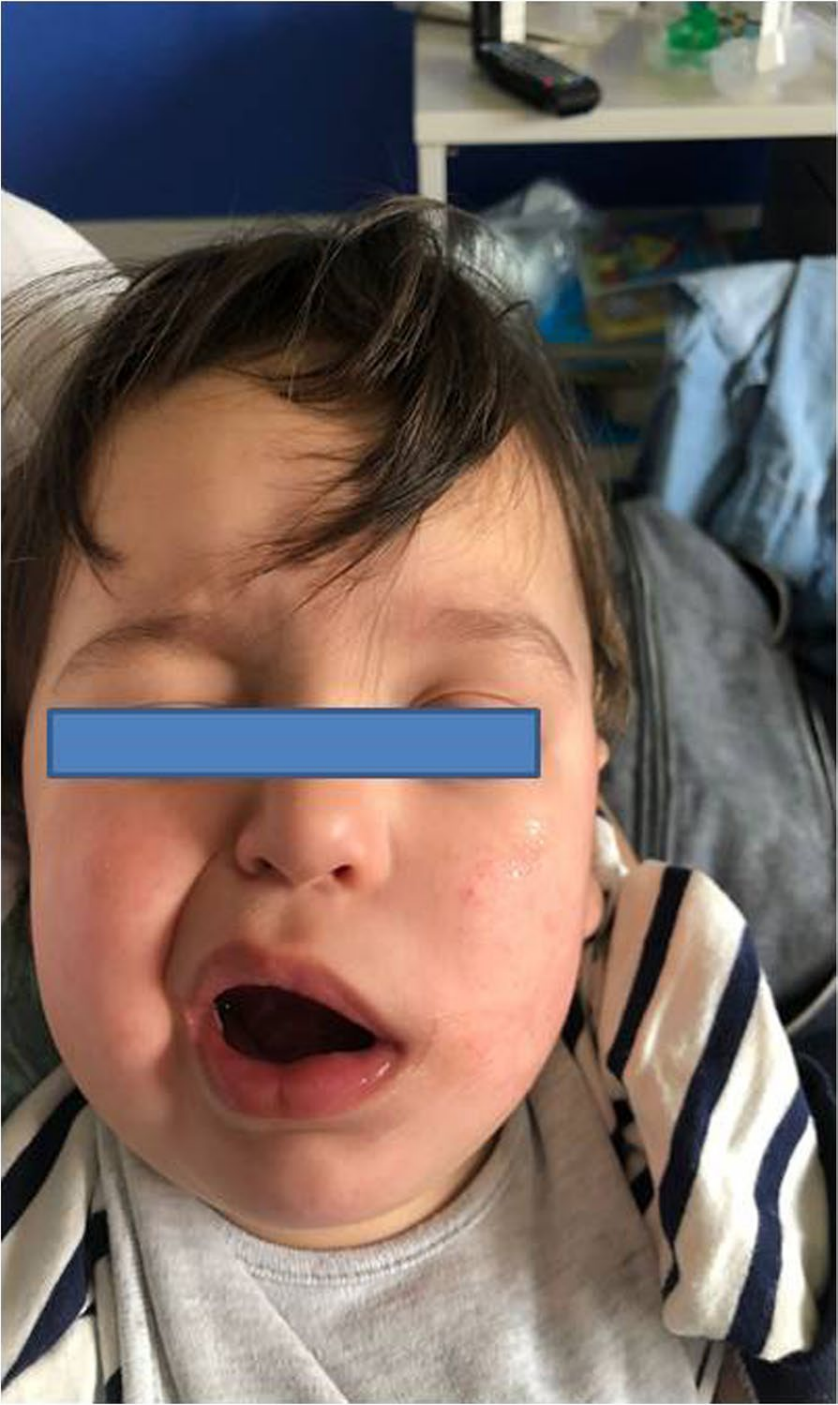
Patient with left facial nerve palsy with incomplete left eye closure at the onset

On admission, his vital signs were: body weight 13 kg; heart rate 106 beats/min; body temperature 38,5 °C; oxygen saturation in room air 98%. The patient was in good general condition, with normal cardiorespiratory and abdominal examinations. No skin rash was observed. There was no sign of meningitis. The neurological examination revealed a loss of the left nasolabial fold, a left facial drop with sparing of the forehead muscles, and ptosis of the left upper eyelid, consisting of a grade IV peripheral left facial nerve palsy with the House-Brackmann scale (Fig. 1).

Otoscopy showed bilateral tympanic hyperemia with evidence of middle ear effusion but no tympanic membrane bulging. No vesicles in the ear canal and no parotid masses were observed. Furthermore, no postauricular swelling or erythema was detected. No auricular displacement or mastoid region tenderness was documented.

Laboratory tests showed a white blood cell count of 13,750/mmc, with neutrophil predominance, and a C-reactive protein of 3,18 mg/dl (normal value < 0.5 mg/dL). A blood bacterial culture was performed and provided negative results.

**Fig. 2:**
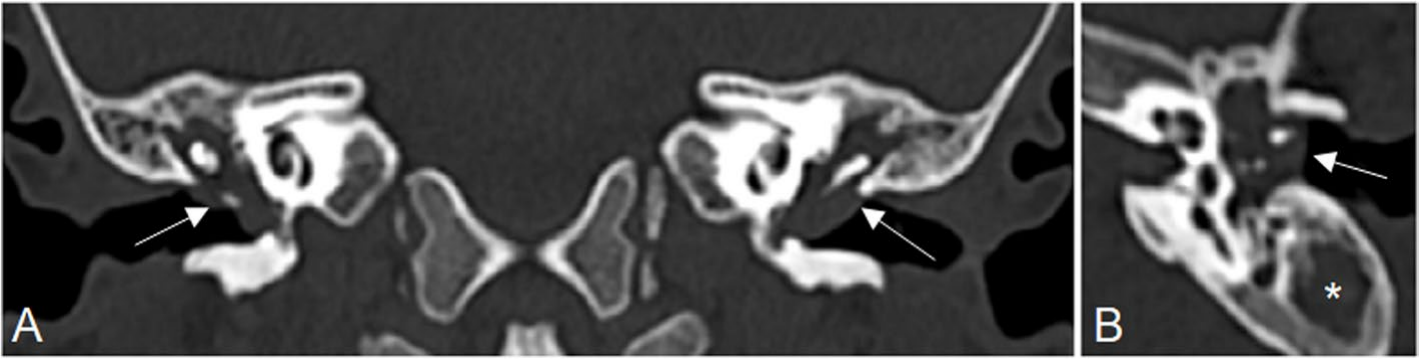
Temporal bone CT scan with (**A**) coronal and (**B**) axial reconstructions shows bilateral middle ear and mastoid effusion with complete opacification of the tympanic cavities (arrow) and mastoid air cells (asterisk)

A computed tomography (CT) scan of the head was promptly performed, which showed bilateral middle ear and mastoid effusion with complete opacification of the mastoid air cells and tympanic cavities without any bony wall erosion neither in the middle ear nor in the external meatus (Fig. 2). The diagnosis of acute AOM-related facial nerve palsy was achieved. The child was immediately started with intravenous cefotaxime (100 mg/kg/day), intravenous antiviral therapy with acyclovir (30 mg/kg/day in 3 doses), and oral steroids with betamethasone (0,2 mg/kg/day), which was progressively tapered off and discontinued within 15 days.

To protect the left eye from corneal damage, artificial tears were applied numerous times a day along with ophthalmic lubricating ointment and an eyelid patch at night. To identify infectious causes of peripheral facial nerve palsy, serologic tests for herpes simplex virus, varicella virus, human herpes virus type 6, coxsackie virus, adenovirus, and *Borrelia burgdorferi* were performed and resulted negative; consequently, antiviral acyclovir was discontinued. Furthermore, a primary EBV infection was serologically detected, which resulted positive for anti-VCA IgM whilst negative for anti-VCA IgG, anti-EA, and anti-EBNA.

**Fig. 3:**
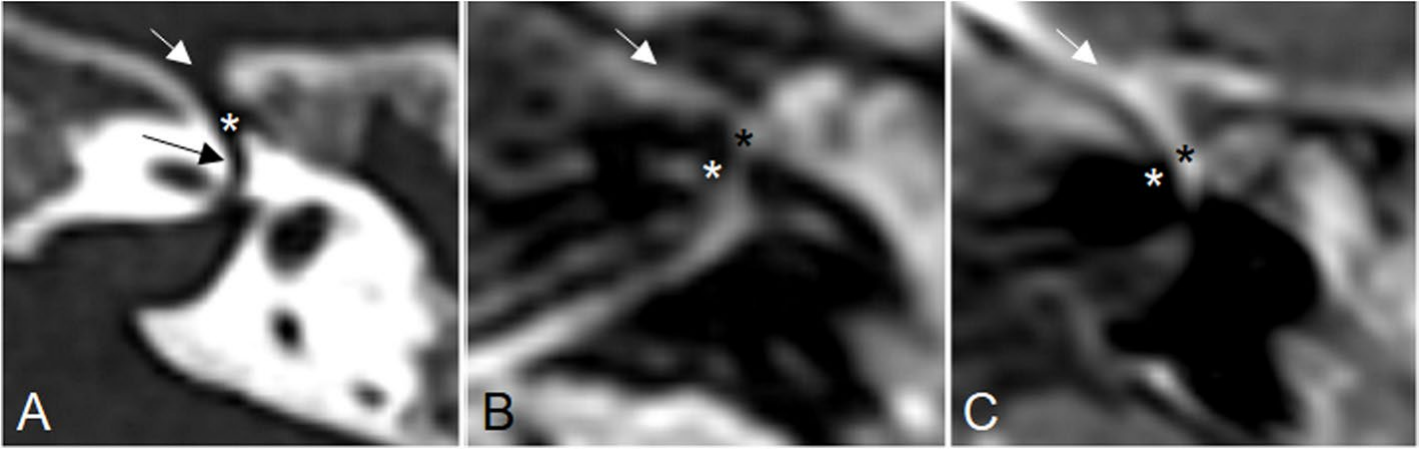
Left temporal bone (**A**) CT and (**B**, **C**) MR axial plane images. **A** The CT image is showed for anatomic reference (black arrow: bony canal of the labyrinthine segment of the facial nerve; white arrow: bony canal of the greater petrosal nerve; asterisk: geniculate ganglion fossa). The MR images show (**B**) slight FLAIR hyperintensity and subtle (**C**) T1 contrast enhancement of the geniculate ganglion (black asterisk), labyrinthine portion of facial nerve (white asterisk), and proximal tract of the greater petrosal nerve (white arrow)

On day 4, a Three Tesla magnetic resonance imaging (MRI) of the head confirmed the presence of middle ear and mastoid effusion, and showed the presence of a slight FLAIR hyperintensity together with a subtle enhancement of the geniculate ganglion and labyrinthine portion of facial nerve indicating an inflammatory reaction (Fig. 3). On day 5, a left myringotomy with placement of a ventilation tube was performed. The bacterial culture of the purulent drainage resulted negative. The patient progressively recovered from the paralysis and was completely apyretic after 2 days of hospitalization. The antibiotic treatment with intravenous cefotaxime was continued for a total of 15 days; afterwards, the patient was discharged. Caregivers were instructed to keep the ear dry, and the child was trained to perform facial muscles gymnastics and tactile stimulation under the supervision of our speech therapist.

On follow-up examination after a few days from discharge, the patient developed a picture of herpangina with pharyngeal hyperemia and vesicles in the oral cavity without a worsening of facial paralysis. After 3 months, the facial paralysis was completely recovered.

## Discussion and conclusions

The facial nerve is responsible for facial expression, lacrimation, and salivation. It emerges from the brainstem at the cerebellopontine angle; then, through the fallopian canal, it enters the petrous temporal bone via the internal auditory meatus; and finally, it exits through the stylomastoid foramen, becoming the extracranial facial nerve [7]. In this phase, the facial nerve forms 5 terminal branches supplying the motor function of the face [8]. Interestingly, the frontal branch has bilateral cortical input, which means that the motor function of the forehead is preserved in upper motor neuron lesions but absent in lower motor neuron lesions [7].

Understanding the anatomy of the facial nerve is essential to identify the level of the lesion in case of facial palsy. Facial palsy may be distinguished from facial nerve palsy (due to a peripheral lesion) or central facial palsy (due to a central lesion involving the upper motor neuron). They clinically differ in the preservation of function of the forehead muscles in the central form, which requires a prompt evaluation for the intracranial process [9].

On physical examination, the patient with facial nerve palsy is unable to raise the eyebrow or close the eyelid on the affected side. The nasolabial fold is typically absent and the affected side presents a dropping mouth rim, with possible saliva leakage and the inability to smile [10]. If stapedius muscle paralysis is present, the patient may experience hyperacusis.

Importantly, lacrimal and salivary production can be reduced, and lagophthalmos can promote corneal irritation with abrasion and ulceration. Therefore, the use of eye-protective measures is strongly recommended [9].

The possible etiologies of facial nerve palsy in children can be divided into congenital or acquired causes, which in turn can be further classified as infectious, traumatic, malignancy-associated, hypertension-associated, and idiopathic (Bell's palsy). Table 1 summarizes the possible causes of facial nerve palsy. The degree of facial nerve palsy can be scored using the House-Brackmann scoring system, as shown in Table 2 [11].

**Table 1:** Etiologies of facial nerve palsy in pediatric age

**Congenital causes**
1. Birth trauma
2. Genetic syndrome • Fascioscapulohumeral dystrophy • Melkersson-Rosenthal syndrome • Osteopetrosis (Albers-Schönberg disease) • Goldehar syndrome
**Acquired causes**
1. Idiopathic (Bell’s palsy)
2. Infectious disease • Bacterial infection: Lyme disease (*Borreliaburgodorferi*), Mycoplasma pneumoniae, Haemophilus influenza, other • Virus infection: Herpes zoster (Ramsay Hunt syndrome), Epstein-barr virus, Cytomegalovirus, Adenovirus, Human herpesvirus type 6, coxsackie virus, SARS-CoV-2, other • Acute otitis media and Acute mastoiditis
3. Inflammatory disease • Henoch-Schönlein purpura • Kawasaki disease • Guillan-Barrè syndrome
4. Neoplastic disease • Posterior fossa tumors • Facial nerve scwannoma • Parotid gland tumors • Leukaemia and Lymphoma
5. Traumatic • Temporal bone fracture • Iatrogenic
6. Metabolic diseases • Diabetes mellitus • Hyperparathyroidism • Hypothyroidism
7. Other • Hypertension, autoimmune diseases…

**Table 2:** House-Brackmann facial nerve grading system

Grade	Description	Clinical characteristics
I	Normal	No paresis
II	Mild paresis	No abnormalities at rest
III	Moderate paresis	No deformity at rest. Facial synkinesis. With maximum effort the patient can totally close eyelids
IV	Moderate-severe paresis	Obvious asymmetry. Facial synkinesis. With maximum effort the patient cannot completely close eyelids
V	Severe paresis	Obvious asymmetry at rest (ptosis of labial commissure, disappearance of the nasolabial fold). Incomplete eyelid closure. Asymmetry in mouth motion
VI	Complete paralysis	Atony at rest. No facial function

Herein, we describe a case of AOM-related facial nerve palsy whose incidence has become extremely rare after the widespread use of antibiotics [12]. The etiology of facial nerve palsy in patients with AOM is unclear, although different hypotheses have been postulated. Firstly, in the early stage, AOM may cause retrograde infection within the facial nerve canal. Secondly, the presence of inflammatory bacterial toxins may induce peripheral demyelination of the facial nerve. Finally, the spread of the inflammatory process into the mastoid region may provoke inflammation or compression of the facial nerve. Moreover, once a chronic infection occurs, a cholesteatoma may directly compress and erode the facial nerve [8]. Other authors suggest that the insult is caused by the compression and thrombosis of the microvasculature supplying the facial nerve, determining neuritis and nerve palsy [13].

In our patient, an EBV primary infection was serologically documented. EBV belongs to the human herpesvirus family acknowledged to cause neurological complications in 0,5–7,5% of patients with acute infection [14, 15]. Facial nerve palsy with AOM associated with primary EBV infection is rarely encountered in children. Volgelnik et al. described 5 cases of unilateral facial nerve palsy with ipsilateral AOM and primary EBV infection [5] in otherwise healthy children between 17–27 month-old, which received antibiotic treatment and underwent a myringotomy of the affected ear with tympanostomy tube insertion within 24 h after admission. Only one patient underwent mastoidectomy in addition to myringotomy. Interestingly, in situ hybridization analysis of biopsied material showed EBV-specific ribonucleic acid. Complete recovery of the facial nerve was documented in 4 patients, whereas a slight paresis occurred in one patient two years after the hospitalization. Recently, Yamaguchi et al. have reported two cases of 18 and 19-month-old female children with unilateral facial nerve palsy with ipsilateral AOM and primary EBV infection [6]. They received oral corticosteroids and vitamin B12 supplementation, and both recovered after 12 and 6 weeks, respectively.

In our patient, the suffering of the facial nerve at the level of the geniculate ganglion and its labyrinthine portion detected through MRI, in absence of any bony erosion, suggested that acute infectious neuritis of the facial nerve spreading from the middle ear (probably through a microvascular route) was the underlying causative mechanism. Under these circumstances, a concomitant EBV infection could have paved the way to a following bacterial superinfection responsible for both the middle ear and the facial nerve infection.

In a patient with AOM-related facial nerve palsy, a CT scan of the head should be performed to exclude the radiological diagnosis of otomastoiditis and to document the presence of associated intracerebral orextracerebral complications [16]. Furthermore, MRI of the head can be used not only for diagnostic purposes but also to detect rare complications of otomastoiditis such as dural sinus thrombosis [17].

Once diagnosed, AOM-related facial nerve palsy should be adequately treated, with the eradication of the suppurative process as the first goal to achieve [18]. To this aim, the patient should receive a broad-spectrum intravenous third-generation cephalosporin (for example cefotaxime as in our case) as an initial antibiotic regimen, to be eventually adjusted based on the microbiological antibiotic sensitivity in positive cultures [16].

To date, no treatment consensus on AOM-related facial palsy is available. Indeed, the role of surgery, corticosteroids, and antivirals remains controversial. Several authors agree on the need for myringotomy with or without tympanostomy tube placement in cases with otomastoiditis without perforation of the tympanic membrane, suggesting to perform mastoidectomy or, rarely, facial nerve decompression if no improvement is achieved within a few days [13, 19, 20]. Recently, Eeten et al. have described two cases of EBV otomastoiditis complicated by facial nerve palsy that, initially not improved with the medical treatment alone, subsequently recovered after a surgical approach with mastoidectomy plus atticoantrotomy and a transmastoidal surgical decompression of the facial nerve, respectively [21].

Antiviral therapy has been used to treat peripheral facial palsy by considering the potential association with herpes simplex virus infection [22]. However, as the real effectiveness of antivirals’ administration in cases with facial nerve palsy is not fully understood yet, antiviral treatment should be avoided if no infectious cause is suspected [9]. As a consequence, in our case acyclovir was interrupted once herpetic virus serology resulted negative.

As far the use of corticosteroid treatment for facial nerve palsy, despite a few studies are available, it seems that steroids may reduce the time of recovery, especially when administered early in the disease course [7, 9]. To better understand the role of corticosteroids in the treatment of children with idiopathic facial nerve palsy, two randomized, double-blind, placebo-controlled trials have recently been started [23, 24].

As observed in our patient, the prognosis of AOM-related facial nerve palsy is generally good after the appropriate therapy, even though a 6% incidence of residual dysfunction has been described [25, 26], and the recovery of facial palsy usually occurs within 3-months. Furthermore, in the case of infectious etiology and incomplete forms, the recovery may be more quickly [12].

In conclusion, our case highlights a rare complication of AOM and primary EBV infection in children the pediatricians should be aware of. A prompt diagnosis and adequate treatment are essential to achieve a good outcome and to avoid chronic neurological sequelae.

## Data Availability

Data sharing was not applicable to this case report because no datasets were generated or analyzed during the study.

## Abbreviations

AOM: Acute otitis media
CT: Computed tomography
EBV: Epstein-barr virus
MRI: Magnetic resonance imaging
